# Kidney autotransplantation after nephrectomy and work bench surgery as an ultimate approach to nephron-sparing surgery

**DOI:** 10.1186/s12957-018-1338-1

**Published:** 2018-02-20

**Authors:** Martin W. W. Janssen, Johannes Linxweiler, Ines Philipps, Zentia Bütow, Stefan Siemer, Michael Stöckle, Carsten-Henning Ohlmann

**Affiliations:** 1 Department of Urology and Pediatric Urology, University of Saarland, Kirrbergerstr. 6, 66421 Homburg/Saar, Germany; 2 Present Address: Groupe Hospitalier Diaconesse Croix Saint Simon Service d’Urologie, Paris, France

## Abstract

**Background:**

Kidney autotransplantation (KAT) is the ultimate approach for nephron-sparing surgery. It is a rarely used method in renal tumor surgery today as minimal invasive and open techniques for nephron-sparing surgery improve constantly. In this publication, the complication rate and the long-term functional and oncological outcome at a single center are analyzed.

**Methods:**

A prospectively constructed database of patients with renal tumors who underwent renal surgery was retrospectively analyzed to identify patients with KAT and describe surgical and oncological outcomes and to obtain long-term follow-up. Data collection included detailed surgical technique, complications (Clavian-Dindo), and hospital stay, as well as functional and oncological outcome and long-term follow-up.

**Results:**

Between 1976 and 2013, 12 patients (median age 50.5 years) underwent KAT for highly complex renal masses: in five cases for complex renal cell carcinoma (RCC), five cases for complex upper urinary tract carcinoma (UTUC), one case for a renal metastasis, and one case for nephroblastoma. The nephrectomy or nephron-ureterectomy was performed open via a flank or transabdominal. The median surgical time was 360 min (range 270–490 min). Intraoperatively, six cases required blood transfusions (50%). Six patients (50%) developed significant postoperative complications (Clavian-Dindo > 2). In two patients, intermittent hemodialysis for delayed graft function (16.6%) was needed, and in six cases (50%), additional blood transfusions postoperatively were necessary. At discharge from hospital, all patients had functioning grafts. The median hospital stay was 29.5 days (range 18–35).

At follow-up (median follow-up of 83.5 ± 40.7 months), six patients had died (50%)—all with functioning grafts (free from hemodialysis). In five cases, recurrence of primary tumor or metastatic disease was recorded. In four cases, the recurrent carcinoma could be resected; in detail, UTUC in three cases and one partial nephrectomy of the autotransplanted kidney was performed. One patient suffered from bone and lung metastasis. Two patients died finally tumor-related. Five patients (41.6%) are presently alive, without evidence of tumor relapse. One patient developed terminal renal failure requiring hemodialysis 105 months after autotransplantation. One additional patient was lost to follow-up; after 69 months, this patient had a functioning kidney and no evidence of disease-recurrence at the last follow-up. A cumulative number of 1424 months without hemodialysis was gained for these 12 patients.

In the literature to date, most KAT are performed in benign disease, with minor but frequent complication. Here, we report the largest series of KAT for malignant kidney tumors. The complication rates are similar, compared to the recently reported series for benign indications with an improved graft survival rate. Since KAT requires a complex and challenging surgical approach, it should be performed by experienced kidney transplant surgeons.

**Conclusion:**

In very complex cases involving renal tumors and multi-morbidity, patients should be counseled well before KAT is considered. At the same time, KAT should not be abandoned in these very rare cases, especially when a nephron-sparing approach is otherwise not feasible. KAT can maintain renal function and quality of life and extend expectancy of life.

## Background

In patients with organ-confined kidney tumors, nephron-sparing surgery improves life expectancy compared to radical nephrectomy, due to a reduced rate of non-cancer associated mortality while being oncologically safe [1]. Patients with tumor-bearing solitary kidneys in particular benefit from organ-sparing approaches, thereby avoiding the necessity of long-term hemodialysis (HD). A mortality rate as high as 15.6% in Europe and 21.7% in the USA [2] is reported in patients on HD. Every effort should be made to prevent nephrectomy in these patients. Today, in most cases, even in cases of anatomically complex renal masses, nephron-sparing surgery, often minimally invasive, can be performed [3]. Therefore, the number of KAT performed decreased significantly and the technique is an infrequent procedure in Urology today. The more common indication for KAT remains vascular injuries or disorders, or complex ureteral strictures [4–7]. However, even in cases of treatment of benign conditions, the number of KAT procedures is decreasing due to the advancement of minimal invasive surgical techniques. Gordon et al. published the first complete robotic autotransplantation of a case of extensive ureteral loss [8].

In cases of large or complex renal tumors in solitary kidneys, work bench ex vivo resection with consecutive KAT of the reconstructed organ is regarded as the ultimate approach in avoiding permanent loss of kidney function. However, hardly any data is available on the long-term oncological outcomes of this specialized surgical approach for treatment of oncological conditions. So far, results of KAT for mainly benign indications have been published [9].

We aim to contribute to the long-term oncological and functional outcome in KAT for malignant indications.

## Methods

From 1976 onwards, data of every case of renal tumor in the Department of Urology and Pediatric Urology, Homburg/Saar, has been collected in a prospective database after approval of the local ethics committee. This retrospective observational study included every case of KAT for the treatment of a malignancy. Seven patients were identified with highly complex renal masses and five patients underwent KAT with pyelovesicostomy [7] for the treatment of complex upper urinary tract urothelial carcinoma (UTUC) of a solitary kidney. The patients’ epidemiological data (Table 1); the surgical, functional, and oncological results (including operative time, ischaemia time, complications according to Clavian-Dindo classification [10]) (Table 2), and long-term follow-up data (Table 3) are presented in this paper. The renometric score introduced by Ficcara (PADUA) was applied retrospectively to one case with a renal cell carcinoma (RCC) in order to describe the anatomical complexity [11]. In the other 11 cases, there was no or only insufficient radiological material stored or available for grading the complexity of the tumors in terms of PADUA or any other scoring systems. The patients’ General Practitioners or those involved in the upkeep of cancer databases were approached for follow-up data. In every case, the time (in months) of a functional graft, or until death with a functional graft in place, was calculated and added to calculate the cumulative gain of renal function of the study population.

**Table 1:** Patient characteristics and indications for KAT

	Number	Median	Range
Male/female	7/5		
Age (years)	12	50.5	[5–70]
Charlson comorbidity index	12	2.0	[2–6]
ASA physical status classification	12	2	[1–3]
Anatomical situation			
Primary anatomical single kidney	6		
Bilateral tumors	6		
Location of tumor			
Central/renal pelvis	10		
Upper/lower pole	2

**Table 2:** Perioperative and pathological results

	Number	Median	Range
Surgical results			
Perioperative mortality	0		
Total operative time (min)	12	360	[270–490]
Warm ischemia time (min)	12	50	[25–60]
Cold ischemia time (min)	12	195	[160–280]
Hospital stay (days)	12	29.5	[18–43]
Intraoperative complications	6		
Transfusion	6		
Postoperative complications (Clavian ≥ 2)	7		
Postoperative transfusion	6 cases		
Delayed graft function	2 cases intermitted hemodialysis		
Functional outcome			
Serum creatinine preoperatively (mg/dl)	12	1.07	[0.5–1.21]
Serum creatinine (at discharge) (mg/dl)	12	1.17	[0.83–4.29]
Hemodialysis at discharge	0		
Pathology			
Tumor diameter (cm) of renal solid tumors	7	8.25	[7–12]
Tumor diameter (cm) of upper urinary tract urothelial carcinoma (UTUC)	5	1.0	[0.6–3.5]
Histology			
Renal cell carcinoma (RCC)	5		
UTUC	5		
Renal metastasis resection	1		
Nephroblastoma	1

**Table 3:** Long-term functional and oncological outcome

	Number	Median	Range
Follow-up			
Follow-up (months)	12	83.5	[9–477]
Patients deceased during follow-up (months)	6^a^	–	[9–246]
Patients lost to follow-up	1	After 69 months with graft function	
Functional outcome and follow-up			
Pat. needed hemodialysis at last follow-up	1	105 months after surgery due to nephrectomy for local recurrence at the autotransplanted kidney	
Cumulative months without hemodialysis on follow-up, or till death with functioning graft	12	1424 months	
Oncological outcome and follow-up			
Recurrence or metastasis	5 cases		
Tumor-related death	2 cases		
RCC progression	1 case		
RCC or UTUC local recurrence	4 cases	Resection of UTUC in ureter or nephrectomy or partial nephrectomy of autotransplanted kidney	
Patients on systemic therapy	1	Pazopanib for met. RCC	
Disease-free survival (months)		48	[12–72]

^a^All patients died with functioning grafts

The surgical technique generally followed the local best practice custom for radical nephrectomy, aimed at achieving the best possible oncological results. All radical nephrectomies were open surgeries. No laparoscopy cases were recorded. Prior to clamping the renal hilum, the patients were given mannitol and furosemide. The ureter was resected with a bladder cuff in cases of upper urinary tract carcinoma (UTUC).

**Fig. 1:**
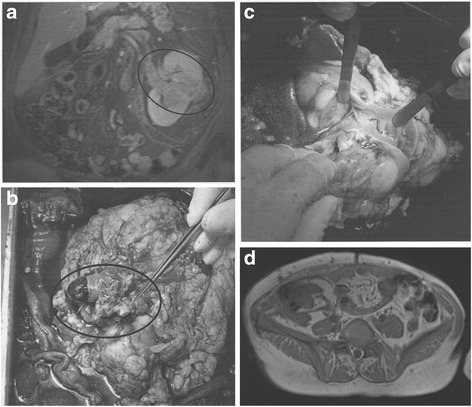
**a** Initial MRI scan PADUA 12p. **b** Tumor thrombus occupying the renal vein. **c** Back-table tumor resection and renal reconstruction. **d** MRI scan showing tumor-recurrence laterally in the autotransplanted kidney in the right iliac fossa. **a**–**d** MRI-images and photographs demonstrating a rare case of an extensive clear cell renal cell carcinoma of the lower pole, extending into the renal hilum and forming a venous tumor thrombus in the inferior vena cava grades PADUA 12p (**a**). A radical nephrectomy, cavotomy, and tumor-thrombectomy were performed (**b**). In ice water, a tumor resection and renal reconstruction was undertaken (**c**) followed by autotransplantation. Local recurrence occurred at 48 months, and a partial nephrectomy of the autotransplanted kidney was undertaken. Finally at 105 months, a nephrectomy was performed for a second recurrence (**d**). At 42 months after nephrectomy, a systemic therapy was introduced for metastases

The tumor-bearing kidney was then perfused back-table with cold storage solution, and the resection of the tumor and reconstruction was performed in iced water (picture series Fig. 1). After a conventional Gibson incision, the kidney was transplanted into the ipsi- or contralateral iliac fossa by means of end-to-side vascular anastomoses to the external or common iliac vessels, with non-absorbable 6–0 sutures. The ureter was implanted into the bladder and stented with a double-J ureteral stent in an extravescial uretereroneocystostomy (modified Lich-Gregory technique). In the UTUC cases, a pyelovesicostomy was performed and stented with a mono-J stent which was placed via the bladder as a percutaneous pyelovesicocutaneostomy.

## Results

The database of the Department of Urology and Pediatric Urology, Homburg/Saar, provided 3765 sets of patient data collected until 2013. Twelve patients (seven male and five female) were identified with complex renal tumors and imperative indications for nephron-sparing surgery, These patients underwent nephrectomy, work bench tumor resection, and kidney autotransplantation. The patients’ characteristics and surgical results are described in Tables 1 and 2. The comorbidity index ranged from 2 to 6 and ASA scoring from 1 to 3. A child of 5 years of age with a nephroblastoma in a single kidney was also included. In all cases, anatomically highly complex malignant renal masses or complex upper tract urothelial carcinomas (UTUC) were identified in single kidneys. In ten cases, the tumors were located centrally or in the renal pelvis, including the five cases of UTUC. In two cases, large tumors of the upper or lower pole with infiltration of the renal hilum were indications for KAT.

During surgery, blood transfusions were necessary in six cases. Postoperatively in seven patients, complications of grade ≥ 2 following the Clavian-Dindo classification occurred. Two patients were required intermittent hemodialysis for delayed graft function, and in six cases, additional blood transfusions were given. No additional surgery was necessary, and after a median hospital stay of 29.5 days, all patients had functioning grafts and there was no indication for additional or permanent hemodialysis. The pathology results for renal tumors showed a median tumor diameter of 8.25 cm and for the UTUC of 1.0 cm. Five of the renal tumors were renal cell carcinoma; one case was a metastatic spread of a different cancer and one case a nephroblastoma.

The exceptional case of one patient suffering from a 8 × 6 × 6 cm large, centrally located tumor in a left solitary kidney was already published in 2007 [12]. A detailed photo-series of this case is presented in Fig. 1.

After 48 months, this patient underwent a partial nephrectomy of the auto-transplanted kidney due to local recurrence of the tumor (Fig. 1d). After a second recurrence, a radical nephrectomy was performed 105 months post-KAT. Histology of the resected kidney described the tumor as pT3a, pNX, L0, V0, Pn0, R0 clear cell RCC. The patient is alive 42 months later, on permanent hemodialysis and on targeted therapy (pazopanib) for an unresectable local tumor relapse. To our knowledge, this patient is the only one of the 12 patients included in our series who required permanent hemodialysis after KAT.

During a median follow-up period of 83.5 months (± 40.7 months), six patients died, all with functioning kidneys and free from hemodialysis at the time of death. One patient was lost to follow-up after returning to his home country after a follow-up of 69 months. At his last follow-up consultation, no recurrence of the tumor was observed. Two patients died of cancer-related conditions after 9 and 77 months. One patient died of a malignant melanoma 187 months after KAT for RCC and one patient of a recurrence of breast cancer after 364 months. Two patients died of non-oncological causes (aortic valve surgery, coronary artery disease). At the 5-year follow-up time-point, only one patient had died for the original cancer. Six of 12 patients (all with RCC) suffered from local or distant tumor-recurrence. Median time to recurrence was 48 months (range 12–72 months). One patient is on systemic therapy (pazopanib) and currently on hemodialysis.

To date, a cumulative figure of 1496 hemodialysis-free months was gained in this study population.

## Discussion

To the best of our knowledge, this is one of the largest published series of KAT used in the treatment of oncological pathology, with long-term follow-up. Since HD reduces quality of life, increases mortality and is an economic burden, work-bench surgery and kidney autotransplantation are generally reserved for desperate situations and often remain the final option to avoid HD in patients who would otherwise require a nephrectomy [5].

Novic et al. reported on KAT in the largest series to date, 108 cases in total which included 14 cases of complex renal cell carcinoma. They reported a primary graft loss of 14% in the 14 cases of renal cell carcinoma. Of the remaining 12 patients in this group, five were free of cancer at follow-up. There was a 5-year OSS of 70%. Only four patients died as a result of RCC recurrence/progression [5]. In 1990, Morgan et al. reported on the hitherto largest series of autotransplantation for renal malignancies only (*n* = 14 patients). These 14 patients had an outcome surgical comparable to that of the series presented in this paper with a 5-year cause-specific survival rate of 54.9 ± 17.2%. It is notable that the overall-survival rate was not different to the enucleation or partial nephrectomy group in their study, which covered data from 1965 to 1987 [13]. They stated that it was important to note that extensive excision or prolonged repair could lead to poor graft function after KAT and that all patients must be counselled appropriately concerning the risk of different interventions and of a high complication rate in the postoperative course.

More recently, Tran et al. reported on 52 patients who underwent autotransplantation after laparoscopic nephrectomy. These included eight patients with malignant renal masses [14]. Of these eight patients, 50% had disease recurrence/progression of which the renal function of two patients could be preserved. They reported a major complication rate (Clavian ≥ 3) of 8 and 12% for early and late complications, respectively.

In our series, only one patient had died due to his original cancer resected in KAT, at the 5-year follow-up time point. Seven of the 12 patients suffered from a grade 2 postoperative complications according to the Clavian-Dindo classification. Blood transfusions were required in six cases. The median duration of the surgical procedure was 360 min (range 310–490 min) and the median hospital stay was 29.5 days (range 18–43 days), which illustrates the high complexity and risk of this procedure. Today, with the advances in minimally invasive and robot-assisted surgery in the field of renal surgery, in situ techniques for highly complex renal masses are preferred and can be performed with good oncological and functional outcomes [15]. Nevertheless, to date, the 1496 months gained in the 12 patients of this study, without HD, seem to counterbalance the risk of this surgical approach. Three of the 12 patients required HD, of which in two cases, it was only temporarily and one started HD 105 months after KAT.

## Conclusion

In very complex cases involving renal tumors and multi-morbidity, patients should be counseled well before KAT is considered. At the same time, KAT should not be abandoned in these very rare cases, especially when a nephron-sparing approach is otherwise not feasible. KAT can maintain renal function and quality of life and extend expectancy of life.
